# Clinical effects of long-term cardiac contractility modulation (CCM) in subjects with heart failure caused by left ventricular systolic dysfunction

**DOI:** 10.1007/s00392-017-1135-9

**Published:** 2017-07-06

**Authors:** D. Müller, A. Remppis, P. Schauerte, S. Schmidt-Schweda, D. Burkhoff, B. Rousso, D. Gutterman, J. Senges, G. Hindricks, K.-H. Kuck

**Affiliations:** 1 Heart and Vascular Center (HGZ), Bad Bevensen, Germany; 2 University Hospital Aachen RWTH, Berlin, Germany; 3 Georg August University of Gottingen, Gottingen, Germany; 4 Columbia University, New York, NY USA; 5 Impulse Dynamics, Hod Hasharon, Israel; 6 Medical College of Wisconsin, Milwaukee, WI USA; 7 Institut für Herzinfarktforschung, Ludwigshafen, Germany; 8 Heart Center Leipzig, Leipzig, Germany; 9 Asklepios Klinik St. Georg, Hamburg, Germany

**Keywords:** Heart failure, CCM, Human, Clinical, Registry, Electrical stimulation, Survival, NYHA, LVEF, MLWHFQ

## Abstract

**Introduction:**

Heart failure is a major cause of morbidity and mortality throughout the world. Despite advances in therapy, nearly half of patients receiving guideline-directed medical therapy remain limited by symptoms. Cardiac contractility modulation (CCM) can improve symptoms in this population, but efficacy and safety in prospective studies has been limited to 12 months of follow-up. We report on the first 2 year multi-site evaluation of CCM in patients with heart failure.

**Methods:**

One hundred and forty-three subjects with heart failure and reduced ejection fraction were followed via clinical registry for 24 months recording NYHA class, MLWHFQ score, 6 min walk distance, LVEF, and peak VO_2_ at baseline and 6 month intervals as clinically indicated. Serious adverse events, and all cause as well as cardiovascular mortality were recorded. Data are presented stratified by LVEF (all subjects, LVEF <35%, LVEF ≥35%).

**Results:**

One hundred and six subjects from 24 sites completed the 24 month follow-up. Baseline parameters were similar among LVEF groups. NYHA and MLWHFQ improved in all 3 groups at each time point. LVEF in the entire cohort improved 2.5, 2.9, 5.0, and 4.9% at 6, 12, 18, and 24 months, respectively. Insufficient numbers of subjects had follow-up data for 6 min walk or peak VO_2_ assessment, precluding comparative analysis. Serious adverse events (*n* = 193) were observed in 91 subjects and similarly distributed between groups with LVEF <35% and LVEF ≥35%, and similar to other device trials for heart failure. Eighteen deaths (7 cardiovascularly related) over 2 years. Overall survival at 2 years was 86.4% (95% confidence intervals: 79.3, 91.2%).

**Conclusion:**

Cardiac contractility modulation provides safe and effective long-term symptomatic and functional improvement in heart failure. These benefits were independent of baseline LVEF and were associated with a safety profile similar to published device trials.

## Introduction

In patients with moderate to severe chronic heart failure and reduced ejection fraction (HFrEF), the mainstay of guideline directed medical therapy (GDMT) includes use of beta-adrenergic blockers, angiotensin converting enzyme inhibitors (ACE-I) or angiotensin receptor blocking (ARB) agents, and aldosterone antagonists. Combination therapy with an ARB and neprolysin inhibitor (LCZ696) may be substituted for the ACE-I/ARB in relevant patients, and Ivabradine is indicated in select subjects with persistent sinus rates over 70 bpm. However, despite optimizing GDMT, up to 50% of patients remain symptomatic with limitation in exertional capacity, and deterioration of NYHA class, exercise endurance, and general well-being [1]. Of these, 35% have prolonged QRS duration or LBBB and are candidates for cardiac resynchronization therapy (CRT) [2]. The remaining 65% have a narrow QRS or RBBB and CRT is not less frequently indicated [3]. For these patients, cardiac contractility modulation (CCM) offers functional improvement, greater exercise tolerance, and symptomatic benefit [4–6]. Recently, CCM therapy was reviewed in the European Society of Cardiology’s guidelines on acute and chronic heart failure (2016) where it was stated that “CCM may be considered in selected patients with HF” [1].

The Optimizer™ system (Impulse Dynamics, Orangeburg, NY, USA), which delivers the CCM therapy, consists of commercially available implantable leads and an externally chargeable impulse generator that delivers non-excitatory biphasic electrical signals to two sites in the RV septum (spaced a few centimeters apart). Impulses are delivered during the absolute refractory period thereby avoiding ventricular capture. When applied this way for 5–12 h/day [4, 7, 8], CCM has been shown to elicit both pathophysiological and clinical benefits. CCM impulse delivery produces an instantaneous enhancement in contractility leading to an acute rise in LVEF over a few hours. This is associated with improved cardiac biochemistry especially in relation to cardiomyocyte calcium handling with upregulation of SERCA-2A, increased phosphorylation of phospholamban, normalization of the sodium-calcium exchanger, and a decrease in BNP [9–11]. These changes are associated with adaptive local remodeling, and a decrease in LVEDP and LVEDD which collectively drive the observed clinical improvement in patients treated with CCM. The clinical benefit includes an increase in LVEF, improved quality of life (Minnesota living with heart failure questionnaire; MLWHFQ), fewer symptoms (NYHA classification), and longer six minute walk test (6 MW), as well as an increase in peak VO [5, 7, 24]. The Optimizer™ system is compliant with available regulations and is commercially available in countries that recognize the CE Mark including the European Union, Russia, Brazil, India, and Australia. Despite substantial clinical experience with over 3000 implants, few reports with small numbers of subjects in specific sites have evaluated the benefit of CCM beyond one year [12–14].

The present registry was established as a means to follow patients originally enrolled in a clinical trial comparing CCM to a control group. Difficulties in recruiting matched control subjects prompted conversion to a prospective registry after 143 subjects had been implanted. The goal was to evaluate long-term (2 years) effects of CCM in each of the 143 symptomatic subjects with HFrEF including several with mid-range ejection fractions (HFmEF). Data acquisition continued until all subjects had completed baseline evaluation and follow-up at 6 months intervals for 2 years (total of 5 evaluations: baseline, 6 months, 12 months, 18 months and 24 months). These data form the basis of this prospective observational report. At baseline and at each interval the impact of CCM on NYHA, MLWHFQ, LVEF, 6 MW, and peak VO_2_ were recorded in accordance with data availability. Data were available at later time points only if the study was performed for clinical indications. As a result, the focus for efficacy data was on NYHA, MLWHFQ, and LVEF since follow-up measurements of 6 MW and pVO_2_ were infrequently obtained. All cause mortality was also determined over the 2 year follow-up period. The present study is the first to report on long-term (2 years) effects (efficacy and safety) of CCM in HFrEF and HFmEF in a large cohort of subjects on a multi-site basis, and is the first to prospectively analyze the benefit of CCM therapy in cases with baseline LVEF below and above 35%.

## Methods

### Patient selection

The CCM-HF investigation included 143 patients with an Optimizer device implanted for clinical heart failure and LVEF <45% between April 15, 2010 (date of first implant) and March 25, 2015 (date of last follow-up visit). The decision to enroll subjects with LVEF >35% was based on the subgroup analysis performed on the FIX-HF-5 study [4, 7] which suggested that patients with LVEF between 25–45% had greater clinical benefit than those in the overall cohort. In that study 35% was used as the upper limit of baseline LVEF based on the site’s evaluation but the core echo lab determined that in 38 patients, LVEF was >35% and these subjects were analyzed separately [7]. For this reason we stratified the patients in the registry according to LVEF (<, ≥35%), allowing us to determine if clinical effectiveness and safety of CCM were similar in subjects with baseline LVEF ≥35% compared to those with LVEF <35%.

### Outcome measures

The following efficacy data were recorded when available: NYHA classification, MLWHFQ score, ejection fraction, peak VO_2_, and 6 min walk distance (6 MW). Safety parameters were recorded including all-cause mortality (primary safety endpoint), cardiac mortality, and rate and severity of related serious adverse events (SAE).

Efficacy data were collected on electronic case report forms, and events were collected by the sponsor, adjudicated and reported. The main efficacy data and all safety data were monitored using an outside vendor. To minimize or avoid bias, the registry involved multiple centers (28 sites in Germany), and site selection was based upon site’s experience with heart failure device implants and availability of an appropriate patient population. The incidence and nature of protocol deviations were evaluated for potential introduction of bias into the data analysis. Every effort was made to follow all subjects to assure the data set was as complete as possible.

### Inclusion and exclusion criteria

Any subject over the age of 18 years who received an Optimizer system implant and provided informed consent was eligible for participation in this registry. Only those subjects who had been taking stable doses of GDMT for at least 30 days were enrolled. There were no exclusion criteria; every patient receiving an Optimizer system implant as part of the originally planned cohort study, could participate. As described above, 143 patients had CCM devices implanted at the time the study was converted to a registry. Only these patients were followed as part of the registry. All patients remained on their initial heart failure medications unless clinical circumstances required a change. There were no restrictions regarding types or doses of heart failure medications used.

### Study procedures and follow-up

Initial baseline measurements included a MLWHFQ questionnaire, echocardiogram, NYHA assessment, pVO_2_, and a six-minute walk test.

The standard implantation protocol of the Optimizer III System used was generally followed. The precordial region of the chest (right subclavian area) was prepped and draped under sterile conditions. After access to the subclavian or cephalic vein, a lead was placed transvenously into the right atrium for sensing atrial activity. Two additional leads were placed transvenously across the tricuspid valve and secured to the right ventricular septum for sensing ventricular activity and bipolar delivery of CCM signals. After recovery from the procedure, a chest X-ray was obtained to exclude pneumothorax and to evaluate lead placement.

The Optimizer™ pulse generator was activated prior to hospital discharge for at least 2 h, while monitoring the subject on telemetry. During this time and device parameters were adjusted as needed and at the end of 2 h, the device was interrogated to ensure proper functioning. At the discretion of the Principal Investigator, subjects were discharged sometime after the 2 h monitoring, having received instructions for recharging the pulse generator including a recommendation to recharge the device weekly. Devices were programmed to be active for an average of 7 ± 1 h/day. A rechargeable battery may help to match device longevity with life expectancy, a problem with most implantable devices [15].

All subjects returned for follow-up between two and four weeks after CCM activation. The pulse generator was interrogated to determine the number of sensed beats, RV lead impedances and the percent of CCM signal delivery (the number of beats actually receiving CCM relative to the total number of ventricular beats sensed during the time period when CCM was programmed to be active). Optimizer parameter settings were adjusted according to the recommendations of the site PI. The patient’s ICD, if present, was also interrogated to insure absence of cross-talk with CCM.

Subjects returned to the hospital for follow-up at 6, 12, 18 and 24 months after baseline assessment. At each visit, the CCM device, and ICD if present, were interrogated to ensure proper functioning and to assess events. An interval medical history, including NYHA classification and medications was obtained. A MLWHFQ, exercise study, and a 6 min walk test were administered if clinically indicated.

At the end of the study period (24 months), the patient and site PI decided whether to maintain the Optimizer in an activated state. If signal delivery continued, follow-up visits were continued accordingly.

### Data validity and statistical analysis

All efficacy data were entered by each site into a common electronic database. Adverse events were reported to the study sponsor and were adjudicated via direct communication with the investigator and reported into a separate database along with efficacy data and measurements. Categorization of serious adverse events (SAE) was done by the site PI and reviewed by the Medical Director. SAEs were categorized as arrhythmic, worsening heart failure, infectious, bleeding, ICD related, Optimizer charging issues, lead problem, death, neurological dysfunction, and renal failure. Cardiopulmonary SAEs outside the above categories were combined under the heading “general cardiopulmonary SAE”, and those related to general medical events not otherwise described above were classified as “general medical SAE”. Validity checks and data cleanup rules were applied with the resulting final data set used for analysis.

Our secondary analysis examined whether the clinical effects of CCM in patients with baseline LVEF ≥35% were no worse than (i.e., is non-inferior to) the clinical effects achieved by patients with initial EF <35%. All data collected were analyzed comparing the follow-up interval results with baseline for the entire cohort as well as between groups, based on baseline LVEF (<35% vs. ≥35%). Data are presented as mean±SD. The significance level used was 0.05.

In addition, analysis of the repeated longitudinal measurements was performed using mixed effects models. Models treated the time point (Baseline, 6 months, 12 months, 18 months, 24 months) as categorically fixed predictors allowing for an arbitrary average time course. Intra-subject correlation was accommodated through a subject-specific intercept and slope. The use of mixed effects models enables robust analysis, despite missing values, based on the totality of available data. In testing for improvement from baseline to follow-up, it was first tested if there is a (global) difference at any of the four follow-up times; if so then changes from baseline to specific time points are tested with allowance for multiple comparisons using Sidak’s method. Comparisons between the baseline LVEF groups were made by including an interaction of the LVEF group indicator and the time variables. These computations were performed using the XTMIXED procedure in Stata 13.

### Ethical considerations

The protocol was developed in accordance with the Declaration of Helsinki and ISO 14,155, and was based on the specific characteristics of the patient population under evaluation.

The study was approved by the Ethics Committee of Leipzig University (Ethik-Kommission an der Medizinischen Fakultät der Universität Leipzig, Institute for klinik pharmacology, Härtelstrasse 16-18, 04107, Leipzig, Germany) and was conducted at 28 sites in Germany.

## Results

One hundred and forty-three (143) patients treated with CCM were followed in this registry. Twenty-eight subjects had baseline LVEF ≥35% (mean 37.3 ± 3.1%). All but one had an LVEF <45%. One hundred and fourteen had LVEF <35% (mean 26.1 ± 5.0%) and one patient did not have a baseline LVEF recorded. This patient’s data is reported in the data analysis for the entire cohort but not in the subgroup analysis by LVEF.

A total of 106 patients completed the follow-up period of 2 years in the registry. The remaining 37 either died or discontinued their participation in the study for other reasons, as detailed below. Results are presented for all 143 patients, except when noted otherwise. Of the thirty-seven (37) patients who did not complete 24 months follow-up, nine (9) patients voluntarily withdrew their consent or were lost to follow-up, ten (10) were withdrawn due to SAE, and eighteen (18) patients died. SAEs and deaths are further discussed below.

Baseline characteristics (mean ± SD) are presented in Table 1. When stratified by baseline LVEF (< or ≥35%), there were no statistically significant differences between the subgroups in any baseline parameter except for the presence of an ICD and minor differences in QRS duration. Thus, the subgroups were well-matched.

**Table 1:** Baseline demographics and characteristics

	All *n* (%)	Group with EF ≥35% *n* (%)	Group with EF <35% *n* (%)
Number of patients	143	28	114
Gender	109 (76%) Male 34 (24%) Female	22 (79%) Male 6 (21%) Female	87 (76%) Male 27 (24%) Female
Age [completed life years]	62 ± 12	65 ± 12	63 ± 12
Subjects with ICD	108 (76%)	16 (57%)*	91 (80%)
Etiology of cardiomyopathy	69 (50%)—Ischemic 57 (41%)—Idiopathic 13 (9%)—other	*N* = 27 16 (59%)—Ischemic 8 (30%)—Idiopathic 3 (11%)—other	*N* = 111 52 (47%)—Ischemic 49 (44%)—Idiopathic 10 (9%)—other
History of CABG and/or PCI	76 (57%)	*N* = 14 (50%)	*N* = 61 (56%)
QRS duration (ms)	118 ± 26 (*N* = 131)	*N* = 24 112 ± 17*	*N* = 106 119 ± 27
NYHA class [Class—N (%)]	II—29 (20%) III—103 (72%) IV—11 (8%)	II—7 (25%) III—21 (75%) IV—0 (0%)	II—22 (19%) III—81 (71%) IV—11 (10%)
Hypertension—N (%)	66 (49%)	*N* = 108 14 (54%)	*N* = 108 51 (47%)
Presence of CRT—N (%)	14 (10%)	2 (7%)	12 (11%)
Cardiac medications	*N* = 133	*N* = 26	*N* = 107
Diuretic	104 (78%)	19 (73%)	85 (79%)
ACE-I	82 (62%)	17 (65%)	65 (61%)
ARB	32 (24%)	8 (31%)	24 (22%)
B-Blocker	126 (95%)	24 (92%)	102 (95%)
Aldosterone inhibitor	87 (65%)	18 (69%)	69 (64%)
Digoxin	19 (14%)	4 (15%)	15 (14%)
Other medications			
Anticoagulation	49 (37%)	8 (31%)	41 (38%)
Antiplatelet Therapy	78 (59%)	20 (77%)	58 (54%)
Statin	92 (69%)	21 (81%)	71 (66%)

For one subject the baseline EF was not known, hence while the entire cohort is of 143 subjects, the total number of subjects in both groups (based on baseline EF) combined, is only 142. * *p* < 0.05 vs. Group with EF< 35%

Using the 3 LVEF stratifications described (EF <35%; LVEF ≥35%; and all subjects combined), functional and quality of life (QOL) characteristics were examined at baseline and throughout the 24 months of CCM therapy.

### NYHA

**Fig. 1:**
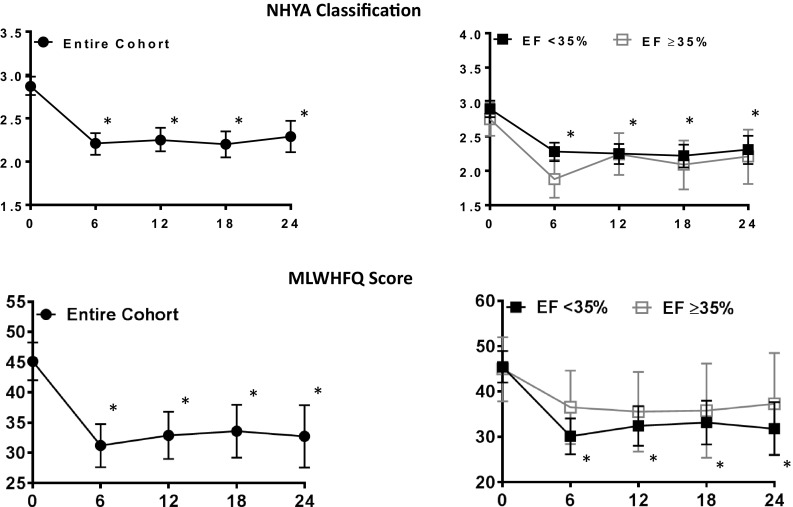
Effect of CCM on NHYA and MLWHFQ. NHYA classification and MLWHFQ both showed sustained improvements over the course of the study. No difference in improvement was seen between LVEF subgroups. * *p* < 0.05 vs. corresponding baseline. Changes from baseline to specific time points are tested with allowance for multiple comparisons using Sidaks method mixed effects models

An improvement in NHYA was observed in overall cohort at each time point during follow-up, compared to baseline (*p* < 0.001), using a mixed effects models analysis (Sidak). A similar and statistically significant improvement in NYHA was seen in the group with LVEF <35% and the group with LVEF ≥35% at each follow-up time point when compared to baseline (Table 2; Fig. 1). The mixed effects models analysis found no statistical difference in the result of the subgroups with baseline LVEF <35% vs LVEF ≥35% (*p* = 0.25 for interaction).

**Table 2:** Impact of CCM on NYHA, MLWHFQ, and LV ejection fraction over time and by EF class

	EF group	NYHA	MLWHFQ		LV ejection fraction	
		Mean (*n*)	Value (*n*)	Δ from baseline	% (*n*)	Δ from baseline
Baseline	EF <35%	2.9 ± 0.5 (114)	45.4 ± 19.6 (104)	–	26.1 ± 5.0 (114)	–
	*EF ≥35%	2.8 ± 0.4 (28)	44.6 ± 17.3 (25)	–	37.3 ± 3.1 (28)	–
	Total	2.9 ± 0.5 (143)	45.0 ± 19.2 (130)	–	28.3 ± 6.4 (142)	–
6 Months	EF <35%	2.3 ± 0.8* (87)	30.0 ± 19.8 (66)	−16.4 ± 20.8*	28.2 ± 8.3 (68)	2.6 ± 7.2*
	EF ≥35%	1.9 ± 0.8* (21)	37.3 ± 18.8 (18)	−9.7 ± 17.9	40.5 ± 6.2 (15)	3.2 ± 6.6
	Total	2.2 ± 0.8* (109)	31.4 ± 19.7 (22)	−15.1 ± 20.3*	30.5 ± 9.2 (83)	2.7 ± 7.1*
12 Months	EF <35%	2.2 ± 0.8* (79)	32.2 ± 21.9 (61)	−12.3 ± 22.8*	28.9 ± 8.8 (62)	3.3 ± 7.8*
	EF ≥35%	2.4 ± 0.8* (19)	35.3 ± 14.5 (15)	−8.9 ± 9.9	39.1 ± 4.3 (17)	2.4 ± 4.7
	Total	2.2 ± 0.8* (99)	32.8 ± 20.6 (76)	−11.6 ± 20.9*	31.7 ± 13.1 (79)	3.1 ± 7.3*
18 Months	EF <35%	2.2 ± 0.7* (70)	32.5 ± 24.3 (59)	−13.0 ± 25.6*	31.1 ± 10.3 (55)	5.3 ± 9.8*
	EF ≥35%	2.1 ± 0.6* (15)	35.0 ± 16.0 (11)	−4.8 ± 15.9	39.3 ± 4.9 (11)	2.4 ± 5.7
	Total	2.2 ± 0.7* (86)	32.9 ± 23.1 (70)	−11.7 ± 24.5*	32.0 ± 10.5 (66)	4.8 ± 9.3*
24 Months	EF <35%	2.2 ± 0.9* (52)	30.8 ± 23.6 (44)	−15.0 ± 21.6*	33.0 ± 9.1 (37)	7.5 ± 9.3*
	EF ≥35%	2.3 ± 0.7* (15)	34.5 ± 18.7 (14)	−9.4 ± 18	40.2 ± 5.6 (13)	3.5 ± 6.0
	Total	2.2 ± 0.8* (68)	31.2 ± 22.5 (59)	−13.6 ± 20.6*	34.9 ± 8.8 (51)	6.5 ± 8.7*

All data are presented as mean ± SD; n’s reflect numbers of subjects with available data. LV ejection fraction (EF; mean±SD). Means and standard deviations of available raw data are shown. *P* values at individual time points were determined by the mixed model using Sidaks method for multiple comparisons. *p < 0.05 vs. corresponding baseline

### MLWHFQ

The impact of CCM on MLWHFQ is shown in Table 2 and Fig. 1. Baseline MLWHFQ scores were similar in all three LVEF groups. The overall group improved their scores significantly at 6-months and sustained the improvement thereafter with a mean improvement of 13.9 at 6-months; 12.2 at 12-months; 11.6 at 18 months; 12.4 at 24-months (all *p* < 0.001). The improvement was of similar magnitude in the two LVEF groups (*p* = 0.58 for interaction) although statistically significant improvement in MLWHFQ from baseline was observed in the LVEF <35% and not in the LVEF ≥35% group on a per-time-point *t* test, likely due to the lower number of subjects in the higher LVEF group.

### LVEF

**Fig. 2:**
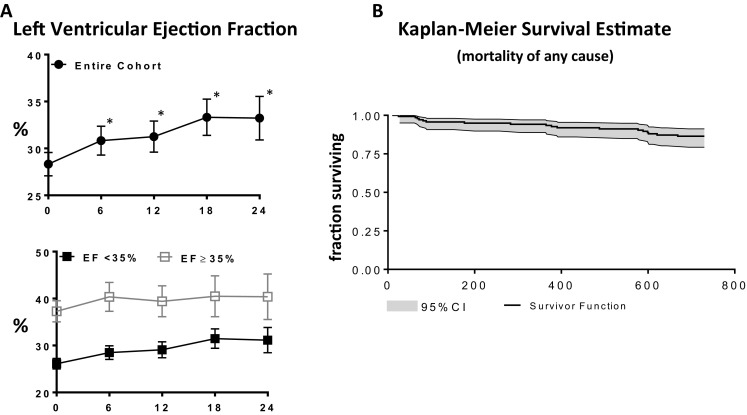
Effect of CCM on LV ejection fraction and all cause mortality. **a** An improvement in LVEF was observed at 6 months compared to baseline and was sustained for 24 months follow-up. Improvements in LVEF were similar between LVEF subgroups. **b** Kaplan–Meier Survival curves for all-cause mortality over the 2 year follow-up. Data are presented as survival function together with 95% confidence limits. *p < 0.05 vs. corresponding baseline. Changes from baseline to specific time points are tested with allowance for multiple comparisons using Sidaks method mixed effects models

Table 2 and Fig. 2 show the changes in LVEF over the course of the study. In the overall group a statistically significant increase in ejection fraction was observed at all time points with an estimated mean improvement in LVEF of 2.5% at 6-months, *p* = 0.003; 2.9% at 12-months, *p* = 0.001; 5.0% at 18-months, and *p* < 0.001; 4.9% at 24-months, *p* < 0.001. The mixed effects model analysis found a similar improvement in LVEF at each follow-up time point between subgroups (baseline LVEF <35% vs LVEF ≥35%; *p* = 0.83 for interaction.

### Peak VO_2_ and 6 min walk distance

Only about a third of the subjects had baseline peak exercise studies performed and no more than 10 had measurements at the 12, 18 and 24 month time points. Fewer than 50 subjects completed the 6 min walk distance at each follow-up time point, rendering the dataset underpowered for adequate statistical comparison.

The efficacy of medical therapy for heart failure may be influenced by the etiology of cardiac dysfunction [16] although not in all cases [17]. We examined the efficacy of CCM in the 69 subjects with ischemic heart disease compared with those with dilated cardiomyopathy. Baseline values for NYHA (2.9 ± 0.5—Isch; 2.8 ± 0.6—DCM), MLWHFQ (46.8 ± 19.4—Isch; 45.7 ± 17.3—DCM), and LVEF (29.1 ± 6.9%—Isch; 27.7 ± 6.0—DCM) were comparable between groups. The improvement over time in each group was likewise similar (data not shown). Thus, improvement in functional and symptomatic parameters with CCM is not dependent upon whether the heart failure is idiopathic or of ischemic etiology.

Implantation of other devices during the follow-up period could have influenced clinical responses to therapy. However, very few such devices were implanted during the course of the 2 year study. Between 6 and 12 months follow-up, 1 patient received an ICD and another patient received a CRT-D. In both cases the implantation was a revision or replacement of an existing device. Two patients received a new ICD device, one between 12 and 18 months, and one between 18–24 months. All patients receiving new or revised devices were in the EF <35% group. Eliminating these patients from analysis did not change the interpretation of the results.

To determine whether improvements in functional class, quality of life, and EF might have been associated with increased use of heart failure medications (ACE-I/ARB, beta-blocker, aldosterone antagonist) we evaluated usage of these medications (initiation, termination, or maintenance) over the course of the study. Results of this analysis are shown in Table 4. The data demonstrate that few patients initiated or stopped heart failure medications over the 2 year follow-up period. Among those who did change their medical regimen, similar numbers started and stopped the medication. For each medication class (beta blockers, ACE-I/ARBs, and aldosterone antagonists), and at each time point, 80% or more of patients maintained use of the same heart failure medications that were prescribed at the baseline time point. We were not able to accurately determine changes in doses of each medication class.

### Serious adverse events

Throughout the 24 months of follow-up, one hundred and ninety-three (193) serious adverse events were reported in ninety-one (91) patients (Table 3). Of these, thirty-two (32) SAEs in twenty-five (25) patients were classified by the investigator as definitely or possibly related to the device and twenty-seven (28) SAEs in twenty (20) patients as definitely or possibly related to the procedure. In view of overlap between events reported as device related and procedure related, in the aggregate there were thirty-four (34) device and/or procedure related SAEs reported in twenty-five (25) patients during the study period, most commonly due to lead migration. SAEs are presented in Table 3, stratified by baseline ejection fraction (<35% vs. ≥35%).

**Table 3:** Summary of reported Serious Adverse Events

Category	All (*N* = 143)		EF ≥35% (*N* = 29)		EF <35% (*N* = 113)	
	Events	Patients (%)	Events	Patients (%)	Events	Patients (%)
Arrhythmia	20	14 (10)	3	3 (10)	17	13 (12)
General cardiopulmonary	30	23 (16)	3	23 (10)	27	20 (17)
Worsening heart failure	55	37 (26)	11	6 (21)	44	33 (29)
Infection	16	14 (10)	3	3 (10)	13	11 (10)
Bleeding	5	4 (3)	1	1 (3)	4	3 (3)
ICD related	5	5 (3)	1	1 (3)	4	4 (4)
Optimizer IPG malfunction	5	5 (3)	2	2 (7)	3	3 (3)
Lead migration/revision	12	10 (7)	4	3 (10)	8	7 (6)
General medical	41	28 (20)	6	5 (17)	35	23 (20)
Death—unknown cause	4	4 (3)	–	–	4	4 (4)
SAE probably or possibly related to device	32	25 (17)	6	5 (17)	26	20 (18)
Total	193	91 (64)	34	17 (59)	159	74 (65)

Arrhythmia includes: supraventricular tachyarrhythmia (atrial fibrillation, atrial flutter, supraventricular tachycardia, ectopic atrial tachycardia), VT, and VF. General cardiopulmonary includes: angina, dyspnea, pericardial effusion/tamponade, pulmonary related (except pneumonia), syncope, venous thromboembolic disease, and valvular disease. Infection includes: ICD pocket infection, optimizer pocket infection, pneumonia, and sepsis. General medical includes: renal failure, neurological dysfunction, peripheral arterial disease/event, stroke, and other non-cardiac medical abnormalities. SAE’s probably or possibly related to the device are included in the total values

Ten (10) patients were withdrawn from the study due to an SAE after a mean time of 338 days. Of these, 4 events were classified by the investigators as related or possibly related to the device and/or procedure: infection in the ICD pocket (although not in the Optimizer pocket), Optimizer IPG removal during a CRT implantation, hematoma in IPG pocket, and IPG pocket infection.

The thirty-two serious adverse events related or possibly related to the device occurred in 17% of the total study population over the study period: 17% of those with LVEF >=35%, and 18% of those with LVEF <35%. The most common of these SAEs was lead migration. During the two year period, 171 hospitalizations (all cause) occurred.

### Deaths

The primary safety end-point of death of any cause occurred in 18 enrolled subjects during the 24 month follow-up period (average time from enrollment was 341 ± 240 days (range 27–659 days). Three (3) of these were among the 29 patients with baseline LVEF ≥35%. Of the 18 who died, 7 deaths were classified as cardiovascular, 8 were non-cardiovascular, and in three it was not known. None of the deaths were classified by investigators as being related to the device or to the procedure. Kaplan–Meier analysis of survival estimate for all patients in this study through 2 years is shown in Fig. 2. The survival proportions and 95% CIs were 94.2% (88.8, 97.1%) for 1 year, and 86.4% (79.3, 91.2%) for 2 years.

## Discussion

This study represents the largest long-term (24 month) efficacy and safety evaluation of heart failure patients implanted with an Optimizer device. Prior randomized trials followed patients for 3, 6 and 12 months [4, 7]. There are 2 key new findings. First, efficacy and safety of CCM are observed in patients with symptomatic heart failure for at least 2 years. Second, when patients are stratified by baseline LVEF (<35 or ≥35%), both groups demonstrated a similar improvement in NYHA classification, MLWHFQ and LVEF at 6, 12, 18, and 24 month follow-up time points. The rates of SAEs and death were comparable between groups and consistent with prior studies.

Cardiac contractility modulation is known to be effective and safe in treating patients with chronic heart failure with ejection fractions below 35% [4, 6, 7, 18]. Secondary analysis of data from the FIX-HF-5 sub-study suggests that the efficacy of CCM is maintained and possibly greater in patients with LVEF between 25% and 45% [4]. In the present study, similar improvements in efficacy were observed when the prospectively defined analysis was stratified by baseline LVEF above and below 35%.

The importance of evaluating CCM efficacy in these patients with less severely reduced ejection fractions is supported by recently published long-term mortality and hospitalization data suggesting a long-term improved survival in patients with LVEF between 25–40% compared to those with LVEF <25% who are treated with GDMT+CCM vs. GDMT alone [14].

NYHA symptoms improved by 0.70 points within 6 months in the entire cohort with similar changes in each subgroup. This represents a significant improvement on par with or greater than prior studies involving CCM where subgroup stratification occurred at LVEF = 25% [19]. In the present study the markedly improved NYHA score of 0.7 was maintained throughout the 2 years of treatment.

Quality of life, assessed with the validated MLWHFQ, improved vs. baseline in the entire cohort of patients by 11–15 points throughout the follow-up period of 6–24 months. This compares to FIX-HF-5 study which showed an improvement of 9.7 points beyond that observed in the OMT control group at 12 months [19]. In that study, patients with LVEF ≥25% showed greater improvement than those with LVEF <25%. The present registry demonstrates that although both subgroups improved over time, a trend toward greater improvement was seen in the subgroup with lower LVEF (<35%). The reason for the differences is not clear but the small numbers of patients in the higher LVEF subgroups, study design biases, or the different comparators (randomized controls vs. within-subject baseline values) may be explanatory factors.

Baseline LVEF among all study participants averaged 28.3 ± 6.4% and increased at each time point studied, reaching 34.9 ± 8.8% at 24 months. Similar and significant increases were observed in the subgroup with baseline LVEF <35%. Many fewer subjects (*n* = 13) in the group with LVEF ≥35% had echocardiographic assessment at 2 years follow-up, yet a strong trend toward improvement in LVEF was observed (LVEF = 40.2 ± 5.6%, *p* = 0.055 vs. baseline). Lack of statistical significance likely reflects insufficient power for this parameter. The only prior randomized controlled trial that reported changes in LVEF over time had interpretable echocardiographic information in only one half of subjects randomized and saw no change in control or CCM groups over the course of the 6 month crossover trial [7].

Previous studies with small numbers of subjects have observed improvements in LVEF with relatively short-term CCM. Stix et al. examined the effect of CCM in 23 subjects with NHYA class III CHF followed for 8 weeks [18]. LVEF increased from 22 ± 7% to 28 ± 8% (*p* = 0.0002) over this time. In a separate study of 13 subjects with NYHA III heart failure extending to 24 weeks, Pappone reported an improvement in LVEF during CCM from 22.7 ± 7% to 37 ± 13% (*p* = 0.004) [20]. A single center long-term follow-up by Kuschyk et al [12] showed sustained improvements in LVEF, similarly to the present study. The current study is the first prospective multicenter report of sustained improvement in LVEF in patients with HFrEF and HFmEF treated with CCM. Interestingly, among the 38 patients with LVEF <35% at baseline and who had repeat echocardiography at 24 months, 11 improved their LVEF to >35%. Six of those 11 had improved above the 35% threshold by 6 months. This raises the question of whether CCM added to GDMT could reduce the number of patients with an indication for ICD placement.

Although trends toward improvement were observed, we found no statistically significant improvement in 6 MW or pVO_2_ during follow-up, even though other prospective clinical trials did observe improvement in 6 MW times [7, 21] at shorter follow-up times. Several factors may contribute to the lack of statistically significant improvement in 6 MW, including the small number of subjects from whom data were available (only 41 of 130 completing the study had 6 MW testing, and 7 had pVO_2_ measurements at 24 months), and lack of a control group.

Similar to 6 MW, few subjects completed exercise testing throughout the study. Only 51 performed baseline exercise testing and data from 7 were available at the 24 month follow-up visit. The low participation rate likely relates to the fact that testing was done only for clinical indications since data was obtained as part of a registry. As a result it is not possible to draw any conclusions about the effects of CCM on pVO_2_ in this study. However, this question has been addressed in prior studies [4, 5, 7, 19] which demonstrate an improvement in pVO_2_, especially in subgroups with higher baseline LVEF [4, 6].

In our study, 14 subjects had already received a CRT device (13 with CRT-D and one with CRT-P). For most of these subjects enrollment occurred due to failure of the CRT to improve symptoms. In each case the CRT device was turned off when CCM was implanted. Although the numbers who completed follow-up functional testing are insufficient to determine efficacy of CCM in this subgroup (less than ½ completed 24 month follow-up testing), a prior short-term study indicated that CCM can be effective in patients who fail CRT [22]. Nagele used CCM to treat 16 patients with severe heart failure who failed to respond to CRT [22]. After three months of follow-up, LVEF improved from 28.1 ± 7% to 33 ± 17% (*p* < 0.01) [22].

The risk profile for CCM in this study was comparable to that described previously for patients with HFrEF and was primarily related to issues with lead malfunction. In patients with LVEF ≥35%, SAEs were observed in 59% of patients after 2 years compared to 38% at 12 months. Similarly in patients with LVEF <35%, SAE rates were seen in 65% of subjects at 24 months and in 40% of subjects at 12 months. This is comparable to (and potentially lower than) the largest randomized controlled trial of CCM (FIX-HF-5) which reported over a 50 week period, SAEs in 61 and 54% in the study groups [19]. Device related SAEs occurred in 13% of Optimizer treated patients in FIX-HF-5 during the 50 week follow-up, and in 17% of patients in the current study over 2 years. Mortality in the present study was similar to that observed in prior studies, although the number of deaths (*n* = 18 in 2 years) is too small for statistical comparison. For example in a retrospective study in 81 patients [12], Kaplan–Meier curves over a 2 year period paralleled mortality in the present study (Fig. 2). Many baseline patient characteristics were similar between the two studies (age, gender, QRS duration, NHYA class, and heart failure etiology) although LVEF was lower in the Kuschyk study (23.1 ± 7.9%) compared to the present study (28.3 ± 6.4%). Based on all the above, adverse events reported in current study reflect an acceptable safety profile consistent with prior experience using the Optimizer device and commensurate with other implantable devices in a patient population with similar acuity.

Malignant arrhythmia generation is of particular concern in heart failure since this accounts for a large percentage of deaths. Implantation of ICDs has improved survival in this regard. The precise effect of CCM on ventricular arrhythmias has not been directly studied. It is known that application of current, sub-threshold for ventricular capture, applied to the heart during the refractory period can reduce or terminate ventricular tachycardia [23]. Whether CCM evokes similar protection has not been systematically studied although substudy analysis of one clinical trial suggests that CCM has no effect on PVCs or duration of VT [24]. In another report by Pappone et al. [20] of 13 patients followed for 8 weeks, CCM was associated with fewer daily episodes of NSVT and a trend toward a reduction in PVCs. In the largest randomized prospective clinical trial of CCM in heart failure, no increase in ventricular arrhythmias or discharge of ICDs was observed [19]. For patients with an indication for CCM and with symptomatic PVCs, it will be interesting to see if CCM might avert the need for PVC ablation [25]. It would also be of value to examine structural characteristics of the failing heart that might identify super responders, as has been done for CRT [26].

### Study limitations

There are several potential limitations to this study. Significant improvement from baseline over the time course of this study was observed in several subjective metrics related to quality of life and symptoms. We believe these changes to be valid and not substantively influenced by the prominent placebo effect common with device therapy since prior studies involving CCM showed that the initial placebo effect was not sustained beyond 3 months [7] (FIX-HF-4). An objective outcome measure, ejection fraction also improved in both the subgroup with LVEF <35% and in the total cohort at each of the follow-up time points. This further argues against a prominent placebo effect, and supports real and sustainable benefit of CCM therapy.

In a registry, follow-up testing is performed based on clinical need. This factor limited the number of patients available with outcomes data related to LVEF and exercise tolerance including 6 min walk test, and peak VO_2_. Nevertheless, we were able to reliably measure NYHA and MLWHFQ in a large number of subjects through the entire 2 year follow-up period.

Improvement in NYHA, MLWHFQ, and LVEF could have resulted from the increased use of pharmacological treatment of heart failure in these patients. However, analysis of use of heart failure medications (Table 4) reveals that very few subjects initiated or terminated heart failure medicine use over the course of the study. A similar and small number of patients started and stopped specific medications with the majority (>80%) maintaining the same regimen used at enrollment. This analysis suggests that additional medical therapy is not likely the explanation for improvement in measured parameters over the time course of this study. We cannot exclude a change in dosage of heart failure medications as contributing to improved outcomes, but, inclusion criteria required stable use of guideline-directed heart failure therapy for one month prior to enrollment, thus optimal doses were likely already achieved at the beginning of the study.

**Table 4:** Variation in medication use by subjects over the course of the study

	Patients Chronically Treated at Baseline (%)	Patient numbers (% of reported data)			
		Base-6 mo	Base—12 mo	Base—18 mo	Base—24 mo
ACE-I/ARB	112 (84)				
Added		3 (3)	4 (4)	6 (7)	6 (8)
Stopped		6 (6)	7 (7)	7 (8)	7 (9)
No change or not used		97 (92)	93 (89)	80 (86)	62 (83)
# of patients with data	134	106	105	95	77
Beta-blocker	127 (95)				
Added		1 (1)	(2)	2 (2)	2 (3)
Stopped		3 (3)	3 (2)	4 (4)	4 (5)
No change or not used		102 (96)	99 (95)	87 (94)	69 (92)
Aldosterone antagonist	87 (65)				
Added		7 (7)	9 (9)	7 (8)	6 (8)
Stopped		8 (7)	12 (11)	11 (12)	7 (9)
No change or not used		91 (86)	83 (80)	75 (80)	62 (83)

The number of patients with reported data at each timepoint is shown (# of patients with data). The number of patients where drug was added, stopped, or unchanged/not used is shown in columns on the right. There was no difference in medication use from baseline to any of three time points (6, 12, 18, or 24 months). This was true for all three classes of heart failure medications (ACE-I/ARB, beta-blockers, aldosterone antagonists)

Lack of a control group poses limitations on interpretation of findings. Contemporary and comparable controls provide rigor in study design to help avoid interference from unrelated and/or unknown sources that could influence the outcome measures. Without a control group, the analysis of this study compared changes over time to baseline measurements. While this controls for inter-patient variability, it may create bias against the CCM intervention since implicit in the analysis it is assumed that baseline function remains constant over time in untreated patients. In fact, outcome variables tend to get worse over time on GDMT, thus the present study might have underestimated the benefit of CCM in this population.

Future studies should identify key biomarkers to predict optimum responsiveness to CCM. Presence of Cheyne-Stokes respiratory patterns [27], CRP, angiopoetin [28] and other serum markers should be examined in relation to CCM efficacy.

### Summary

In summary, in patients with heart failure with reduced LVEF and persistent symptoms despite GDMT, CCM provides sustained improvement in both cardiac function and QOL. The benefit is present not only in subjects with baseline LVEF <35%, but is also in those with LVEF ≥35%. The extended benefit is not associated with an adverse impact on safety beyond what is expected with implantable devices. Consistent with previous clinical studies, these data suggest that CCM may be beneficial in select patients with heart failure, narrow QRS, and symptoms despite optimal medical management.
